# Changes in motor unit firing pattern are associated with post‐exercise blood pressure response in older untreated but not treated hypertensive adults

**DOI:** 10.1113/EP091981

**Published:** 2024-10-29

**Authors:** Ryosuke Takeda, Tetsuya Hirono, Akito Yoshiko, Shun Kunugi, Masamichi Okudaira, Saeko Ueda, Taichi Nishikawa, Kohei Watanabe

**Affiliations:** ^1^ Laboratory of Neuromuscular Biomechanics School of Health and Sport Science Chukyo University Toyota Japan; ^2^ Research Fellow of Japan Society for the Promotion of Science Tokyo Japan; ^3^ Faculty of Liberal Arts and Sciences Chukyo University Aichi Japan; ^4^ Center for General Education Aichi Institute of Technology Toyota Japan; ^5^ Department of Human Nutrition School of Life Studies Sugiyama Jogakuen University Nagoya Japan; ^6^ Graduate School of Health and Sport Sciences Chukyo University Toyota Japan

**Keywords:** ageing, angiotensin II receptor blocker, blood pressure control, hypertension, neuromuscular system, post‐exercise

## Abstract

This study aimed to determine the difference in motor unit (MU) firing pattern between hypertensive and normotensive individuals, and the relationship between MU firing pattern and post‐exercise blood pressure (BP) response in older individuals. Fourteen older untreated (systolic/diastolic BP (SBP/DBP) ≥ 130/80 mmHg, 76 (5) years), 11 treated hypertensive (78 (4) years) and 14 normotensive (SBP/DBP < 130/80 mmHg, 71 (4) years) individuals were studied. Participants performed ramp‐up exercises until 50% of maximal voluntary contraction (MVC) of knee extension and five MVCs. During the ramp‐up exercise, high‐density surface electromyography signals were recorded and each MU firing rate (FR) and recruitment threshold was assessed. The slope of the linear regression between MUFRs and recruitment thresholds was calculated to assess the MU firing pattern. Pre‐ and post‐exercise blood pressure was measured. Change in (∆)SBP from pre‐ to post‐exercise was greater in treated hypertensive than untreated hypertensive individuals (*P* = 0.026). MUFR was lower in treated hypertensive than untreated hypertensive and normotensive individuals (*P *< 0.001). Although the slope was not significantly different between groups (*P* = 0.294), FRs of larger MUs were lower than those of smaller MUs in treated hypertensive and normotensive individuals (*P *< 0.05) but sustained in untreated hypertensive individuals. The FRs of larger MUs and slope were positively correlated with the ∆SBP only in hypertensive individuals (*r* = 0.768 and 0.715; *P* = 0.044 and 0.020). MUFR was lower in treated hypertensive than untreated hypertensive and normotensive individuals. Furthermore, MU firing patterns were associated with the ∆SBP after exercise in older untreated hypertensive individuals, but this relationship was not observed in treated hypertensive and normotensive individuals.

## Abstract (highlights)

- **What is the central question of this study?**
  Does hypertension status, such as using or not using an anti‐hypertensive medication, affect the motor unit (MU) firing pattern in older individuals?
  Is there a relationship between MU firing pattern and post‐exercise blood pressure (BP) response in older individuals?
- **What is the main finding and its importance?**
  The MU firing rate (FR) was lower in hypertensive individuals using angiotensin II receptor blockers (ARBs) compared to untreated hypertensive and normotensive individuals. FRs of larger MUs were associated with MU firing pattern, and each of those was associated with post‐exercise systolic blood pressure (BP) response in untreated hypertensive individuals. This relationship was not observed in hypertensive individuals using ARBs or in normotensive individuals.

## 1 INTRODUCTION

Exaggerated post‐exercise blood pressure (BP) is one of the risk factors for cardiovascular disease in older hypertensive and normotensive individuals (Costa et al., 2020). Some studies reported that the post‐exercise BP response was more remarkable in hypertensive individuals (Delaney et al., 2010; Greaney et al., 2014), but another study showed no significant difference in the post‐exercise BP response between hypertensive and normotensive individuals (Prakash et al., 2005). Such inconsistent results may be due to the existence of individual differences in the post‐exercise BP response. However, there is a lack of consensus and understanding of the mechanisms of individual differences in the post‐exercise BP response in older hypertensive and normotensive individuals.

BP during exercise and post‐exercise is associated with performed force during exercise (Notay et al., 2018), which is regulated by the neuromuscular system (De Luca & Hostage, 2010). However, because the quantification of neural components has been difficult due to methodological limitations, no study has reported on the relationship between post‐exercise BP and details of neuromuscular systems, for example, the motor unit (MU) firing rate (FR), MU recruitment threshold and their relationships.

High‐density surface electromyography (HDsEMG) has been developed with novel algorithms to measure several (10–40) concurrent MU action potential trains (Farina et al., 2008; Holobar et al., 2009), which are a physiological parameter of neural input from the central nervous system to peripheral muscles. Older individuals had lower MUFR at a lower MU recruitment threshold compared to young individuals (Watanabe et al., 2016). Watanabe et al. suggested that this is due to the collapse in the MU firing pattern in older individuals involving a decrease in the inhomogeneity of muscle fibre types with ageing (Watanabe et al., 2016). Interestingly, a previous study demonstrated a significant decrease in microvascular unit perfusion efficiency during exercise when simulating conditions such as decreased dispersion of active fibres, increased MU size, or reversed MU recruitment sequence (Fuglevand & Segal, 1997). These conditions closely mimic those observed in the ageing process (Brown, 1972; Campbell et al., 1973; Galganski et al., 1993; Kanda & Hashizume, 1989). Thus, to maintain adequate capillary perfusion during exercise, larger MUs may need to be recruited more in older individuals. However, activating the larger MUs also increases intramuscular pressure (Sadamoto et al., 1983), leading to increased peripheral vascular resistance (Osada et al., 2015). Therefore, in addition to larger age‐related alterations in MU firing patterns with a decrease in FRs of smaller MUs (Watanabe et al., 2016), an increase in FRs of larger MUs may lead to an increase in peripheral vascular resistance and BP during and after exercise in older individuals.

It is important to note that many hypertensive individuals have increased angiotensin II, which induces an increase in oxidative stress (Bhatia et al., 2012). A previous review article suggested that increased oxidative stress can cause motor neuron injury and contribute to amyotrophic lateral sclerosis, a devastating neurodegenerative disorder characterized by the death of motor neurons, leading to muscle wasting, paralysis and death (Barber & Shaw, 2010). Thus, hypertension may promote age‐related deterioration in the MU firing patterns compared to normotensive individuals. On the other hand, angiotensin II receptor blockers (ARBs), a popular class of anti‐hypertensive drugs, can reduce oxidative stress (Ogawa et al., 2006) and have a neurotrophic effect on spinal motor neurons (Iwasaki et al., 2002). Therefore, hypertensive individuals using ARBs may recover from age‐related changes in MU firing patterns.

In this context, the study aimed to determine (1) the difference in MU firing patterns between hypertensive individuals with or without ARBs and normotensive individuals, and (2) the relationship between age‐related MU firing patterns and post‐exercise BP response in older individuals. We hypothesized that (1) older hypertensive individuals using ARBs would modulate MU firing patterns compared to untreated hypertensive individuals, and (2) older individuals who have greater age‐related alterations in MU firing patterns and rely more on larger MUs during exercise would show a greater increase in post‐exercise BP response. In contrast, older individuals who have smaller age‐related alterations in MU firing patterns and rely less on larger MUs during exercise would show a lesser increase in post‐exercise BP response. To assess the MU firing patterns, we assessed MUFRs at each recruitment thresholds, as well as calculating the slope and *y*‐intercept of the linear regression between MUFRs and recruitment thresholds. Since MUs exhibit a hierarchical firing pattern based on recruitment thresholds (Watanabe et al., 2016), the slope can describe MU firing patterns well (De Luca & Hostage, 2010), and the *y*‐intercept can be interpreted as the amount of neural input from the central nervous system (Watanabe et al., 2022).

## 2 METHODS

### 2.1 Ethical approval

All participants provided written informed consent. The research ethics committee of Chukyo University approved the study protocol (approved number: 2021–13), and it was conducted in accordance with the *Declaration of Helsinki*.

### 2.2 Participants

Forty‐eight older individuals were recruited at a local health promotion class. The data in the female group were derived from a retrospective analysis of our previous research (Takeda et al., 2022). Participants were divided into an untreated hypertensive group (*n* = 14 (females/males = 11/3), systolic BP (SBP) ≥ 130 mmHg and diastolic BP (DBP) ≥ 80), ARB‐treated hypertensive group (treated hypertensive; *n* = 11 (females/males = 8/3), 5 ARB users and 6 ARB+ calcium channel blocker users) and normotensive group [*n* = 14 (females/males = 10/4), SBP < 130 mmHg and DBP < 80 mmHg) according to the ACC/AHA guidelines (Bakris et al., 2019). Treated‐uncontrolled hypertensive individuals (*n* = 3), isolated systolic individuals (*n* = 3, SBP ≥ 130 mmHg but DBP < 80) and diastolic hypertensive individuals (*n* = 3, SBP < 130 mmHg but DBP ≥ 80) were excluded because the size was too small. Thus, a total of 39 older individuals (females/males = 29/10) participated. Single BP measurements at pre‐exercise on the testing day were used to separate the participants into each group. They were non‐smokers and did not consume alcohol for at least 12 h prior to the start of the experiment. All female participants were not using hormone replacement therapy. Exclusion criteria for this study included a history of cardiovascular, metabolic or neuromuscular disease.

### 2.3 Experimental protocol

**FIGURE 1:**
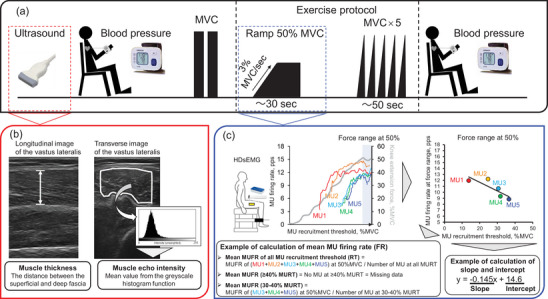
(a, b) Experimental protocol (a) and ultrasound image (b) to assess the muscle thickness and muscle echo intensity and high‐density surface electromyography signals with force signal during the ramp contraction to 50% of maximal voluntary contraction (MVC). (c) Calculation of mean motor unit (MU) firing rate and linear regression analysis of the MU firing pattern; MU firing rate vs. recruitment threshold for vastus lateralis muscle.

Figure 1a shows the experimental protocol. Participants provided two longitudinal images to assess the muscle thickness (MT) of the vastus lateralis (VL), and two transverse images to assess the muscle echo intensity (EI) of VL muscle using ultrasound (Pillen et al., 2009) after height, weight and body mass index measurements. After obtaining ultrasound images, all participants underwent resting BP measurement from the wrist (HEM‐6161, OMRON, Kyoto, Japan) and performed knee extension at maximal voluntary contraction (MVC) twice. Then participants were given an explanation about the procedure of the exercise protocol and practiced several times. First, ramp contraction was applied as submaximal contraction. Individual ramp contractions consisted of a 17 s increasing ramp phase from the baseline to 50% of MVC force levels with an approximately 3% of MVC/s rate of force increase and a 10 s sustained holding phase at 50% of MVC force levels. Participants were provided with visual feedback of the target and performed force via a monitor. If the exerted force did not accurately follow the trace of the target force line, an additional trial was administered at each contraction level. During ramp contractions, HDsEMG signals were recorded from the VL muscle as our group previously described (Watanabe et al., 2016, 2022). Then, five MVCs (10 s rest between each contraction) were performed. Immediately after the exercise protocol, all participants started the post‐exercise BP measurement. It took 1–2 min to complete the BP measurement.

### 2.4 Measurement

#### 2.4.1 Ultrasound images

The participants sat on a bedside, and their knees were fixed at 90 degree flexion in a resting position. Ultrasound images of the VL muscle were obtained using a B‐mode ultrasound device (MicrUS EXT‐1H, TELEMED, Atlanta, GA, USA) with a multifrequency linear array probe (L12‐5L40S‐3). The equipment settings were as follows: frequency of 7.5 MHz, dynamic range of 66 dB, power of −7 dB, gain of 72% and depth of 70 mm. The measurement location was determined at 50% of the distance from the greater trochanter to the upper lateral edge of the patella. An adequate coupling gel was applied to compensate for the depression of the tissues.

Two longitudinal images were acquired while paying attention to placing the probe with minimal pressure and adjusting its angle when the bone echo was brightest and fascia–muscle and bone–muscle boundaries were parallel. Subcutaneous tissue thickness was measured as the perpendicular distance between the surface of the skin and superior fascicle layers. MT of the VL muscle was measured as the distance between the superficial and deep fascia of the VL muscle in longitudinal images (Figure 1b). All thicknesses were determined by the mean value between two longitudinal images.

Two transverse images were also acquired while paying attention to placing the probe with minimal pressure and adjusting its angle when the bone echo was brightest. The transverse images were loaded into software (ImageJ version 1.53k; National Institutes of Health, Bethesda, MD, USA), where the EI of the VL muscle was measured. The region of interest was set as wide as possible, excluding the surrounding fascia and bone. EI was evaluated by the average greyscale value of the region of interest, which was calculated by the standard histogram function (Figure 1b). The grayscale values range from 0 (black) to 255 (white), with a higher value indicating a greater amount of fat and fibrous tissue within the muscle (Pillen et al., 2009). EI of the VL muscle was determined as the mean value between two transverse images.

#### 2.4.2 Blood pressure

BP was obtained from the wrist before measuring MVC, and measurement was started immediately after the exercise protocol and obtained within 1–2 min after the exercise protocol. The wrist BP device is relatively easy and convenient to use at rest and after exercise. The cuff can be more easily wrapped around the wrist than the arm, and undressing is not necessary. However, wrist BP measurement has several limitations of measurement accuracy. In general, the SBP increases in more distal arteries, whereas the DBP decreases (O'Rourke, 2002). Further, the wrist BP measurement depends on whether the location of the wrist is heart level or not (Pickering et al., 2005). Thus, the authors observed very carefully that participants maintain their wrists at heart level during BP measurement.

#### 2.4.3 Maximum isometric voluntary contraction

Participants were seated in a custom‐made dynamometer (Takei Scientific Instruments Co., Ltd, Niigata, Japan) fixed to a force transducer (LU‐100KSE; Kyowa Electronic Instruments, Tokyo, Japan). The hip and knee were flexed at 90 degrees. They performed maximum voluntary isometric contraction involving knee extension twice. The peak force during the contraction was recorded and the greater value of the two measurements was determined as the MVC force. The MVC torque was calculated by multiplying the MVC force and arm length, which was determined as the distance between the knee joint axis and force transducer.

#### 2.4.4 HDsEMG recording

HDsEMG signals were recorded from the VL muscle with a semi‐disposable adhesive grid of 64 electrodes (13 rows and 5 columns with one missing electrode) with a 1 mm diameter and an 8 mm inter‐electrode distance (GR08MM1305, OT Bioelettronica, Turin, Italy) during ramp contraction at 50% MVC, as previously described (Watanabe et al., 2016, 2022). Prior to attaching the electrode grid, the skin was cleaned with alcohol. The longitudinal axis of the VL muscle was identified along the line between the head of the great trochanter and the inferior lateral edge of the patella; the distance between these two anatomical reference points was used as the muscle length. The mid‐point of the longitudinal axis of the VL muscle was measured and marked on the skin. The electrode grid was placed with its centre on the marked position, and the columns were aligned with the longitudinal axis of the VL muscle. The position of the missing electrode was located proximally. The conductive gel was inserted into the cavities of the grid electrode to ensure proper electrode contact with the skin. A wet electrode strap (WS2, OT Bioelettronica) was placed at the knee. Monopolar surface electromyography (EMG) signals were recorded with a band‐pass filter (10–500 Hz), amplified by a factor of 256, sampled at 2000 Hz, and converted to digital form by a 16‐bit analog‐to‐digital converter (Sessantaquattro, OT Bioelettronica). The signal from the force transducer of the dynamometer was also recorded and synchronized with this analog‐to‐digital converter.

### 2.5 Data analysis for HDsEMG signals

During submaximal contractions, individual MUs were identified from the recorded monopolar EMG signals by the convolution kernel compensation technique using DEMUSE software (Holobar & Zazula, 2004, 2008; Holobar et al., 2009). The procedures for decomposition into individual MUs used in the present study were previously and extensively validated based on HDsEMG signals from various skeletal muscles, including the VL muscle (Farina et al., 2010; Gallego et al., 2015; Holobar et al., 2009; Watanabe et al., 2016, 2022; Yavuz et al., 2015). Based on Holobar et al., the pulse‐to‐noise ratio was used as an indicator of the MU identification accuracy, and only MUs with a pulse‐to‐noise ratio >30 dB (corresponding to an accuracy of MU firing identification >90%) were employed for further analysis; all other MUs were discarded (Holobar et al., 2014). The FRs of each MUs were analysed five pulses from reaching the force level at 50% MVC. Several MUFRs identified from one participant were averaged to determine the mean MUFR in each participant. The MUFR and mean MUFR were further calculated for four groups divided by the recruitment thresholds (<20% MVC, 20–30% MVC, 30–40% MVC and 40–50% MVC). Briefly, when five MUs were detected from one participant, the mean MUFR of all MU recruitment thresholds was calculated from the average FRs of the five MUs. When three out of five MUs were detected between 30% and 40% MU recruitment thresholds in one participant, the mean MUFR at 30–40% MU recruitment thresholds was calculated from the average FRs of these three MUs (Figure 1c).

The MU firing pattern was assessed using the slope and *y*‐intercept of the linear regression between FRs of individual MUs and recruitment thresholds (Figure 1c). To assess the age‐related alterations in the MU firing pattern, a linear regression analysis was performed when the number of detected MUs was five or more in each participant (Colquhoun et al., 2018).

### 2.6 Statistical analyses

Values are expressed as means (standard deviation). A mixed analysis of variance with repeated measures model was used to evaluate SBP and DBP (group × time) and a two‐way analysis of variance was used to evaluate MUFR or mean MUFR at each recruitment thresholds (group × MU recruitment threshold) since the normality test was passed. Other variables between the untreated hypertensive, treated hypertensive, and normotensive groups were compared using a one‐way analysis of variance if the normality test was passed. Bonferroni‐corrected *post hoc* procedures were used when applicable. If the test failed, we used the Kruskal–Wallis test to compare variables between the groups. To assess the effect size of mean MUFR and slope, Cohen's *d* was used. Pearson's correlation was used to determine the association of slope and mean MUFR, and change in (∆) SBP or ∆DBP from pre‐ to post‐exercise with mean MUFR, slope, *y*‐intercept, MVC, MT and EI if the normality test was passed. If the test was failed, we used Spearman's correlation. A *P*‐value of <0.05 was considered significant. Statistical analyses were performed using IBM SPSS Statistics v. 25 (IBM Corp., Armonk, NY, USA).

## 3 RESULTS

### 3.1 Participant characteristics

Table 1 shows participant characteristics. Age was significantly higher in the untreated and treated hypertensive groups than in the normotensive group (*P *< 0.001). Body mass index (BMI) was significantly higher in the treated hypertensive than in the normotensive group (*P* = 0.029). SBP and DBP at pre‐exercise were significantly higher in the untreated hypertensive group than in the treated hypertensive and normotensive groups (both *P *< 0.001). SBP at post‐exercise was significantly higher in the untreated hypertensive group than in the normotensive group (*P *< 0.001). DBP at post‐exercise was significantly higher in the untreated hypertensive group than in the treated hypertensive and normotensive groups (*P* = 0.025). There were no significant differences in other parameters, including knee extension strength (*P* = 0.436) and muscle components, MT (*P* = 0.965), or EI (*P* = 0.663), between groups.

**TABLE 1:** Participant characteristics.

Characteristic	Untreated hypertensive	Treated hypertensive	Normotensive	*P*‐value
Number of participants, F/M	11/3	8/3	10/4	—
Age (years)	76 (5)^a^	78 (4)^a^	71 (4)	<0.001 (a)
Height (cm)	152.3 (6.4)	156.4 (6.7)	156.2 (7.2)	0.186 (a)
Weight (kg)	53.8 (9.9)	57.7 (7.3)	52.1 (7.9)	0.274
Body mass index (kg/m^2^)	23.1 (2.6)	23.5 (1.8)^a^	21.3 (1.8)	0.029
SBP at PRE (mmHg)	150 (17)^a,b^	122 (15)	115 (9)	<0.001
DBP at PRE (mmHg)	93 (12)^a,b^	71 (8)	75 (8)	<0.001
SBP at POST (mmHg)	147 (21)^a^	137 (22)	122 (13)	<0.001
DBP at POST (mmHg)	89 (14)	78 (9)	79 (7)	0.025
MVC (N m)	85 (35)	95 (44)	95 (24)	0.436 (a)
Muscle thickness (cm)	2.0 (0.5)	1.9 (0.7)	2.0 (0.5)	0.965
Muscle echo intensity (a.u.)	52.6 (12.1)	55.8 (14.1)	57.0 (13.0)	0.663
Antihypertensive treatment				
ARB	0	5	0	–
ARB + CCB	0	6	0	–

*Note*: Data are means (standard deviation). One‐way analysis of variance was used to compare the groups if normality and equality tests were passed. If those tests failed, the Kruskal–Wallis test was used. (a) Indicates the *P*‐value from the Kruskal–Wallis test. ^a^Significantly different vs. the normotensive group. ^b^Significantly different vs. treated hypertensive group. *P* < 0.05. Abbreviations: a.u., arbitrary units; ARB, angiotensin II receptor blocker; CCB, calcium channel blocker; DBP, diastolic blood pressure; F/M, females/males; MVC, maximal voluntary contraction; SBP, systolic blood pressure.

### 3.2 BP response from pre‐ to post‐exercise

**FIGURE 2:**
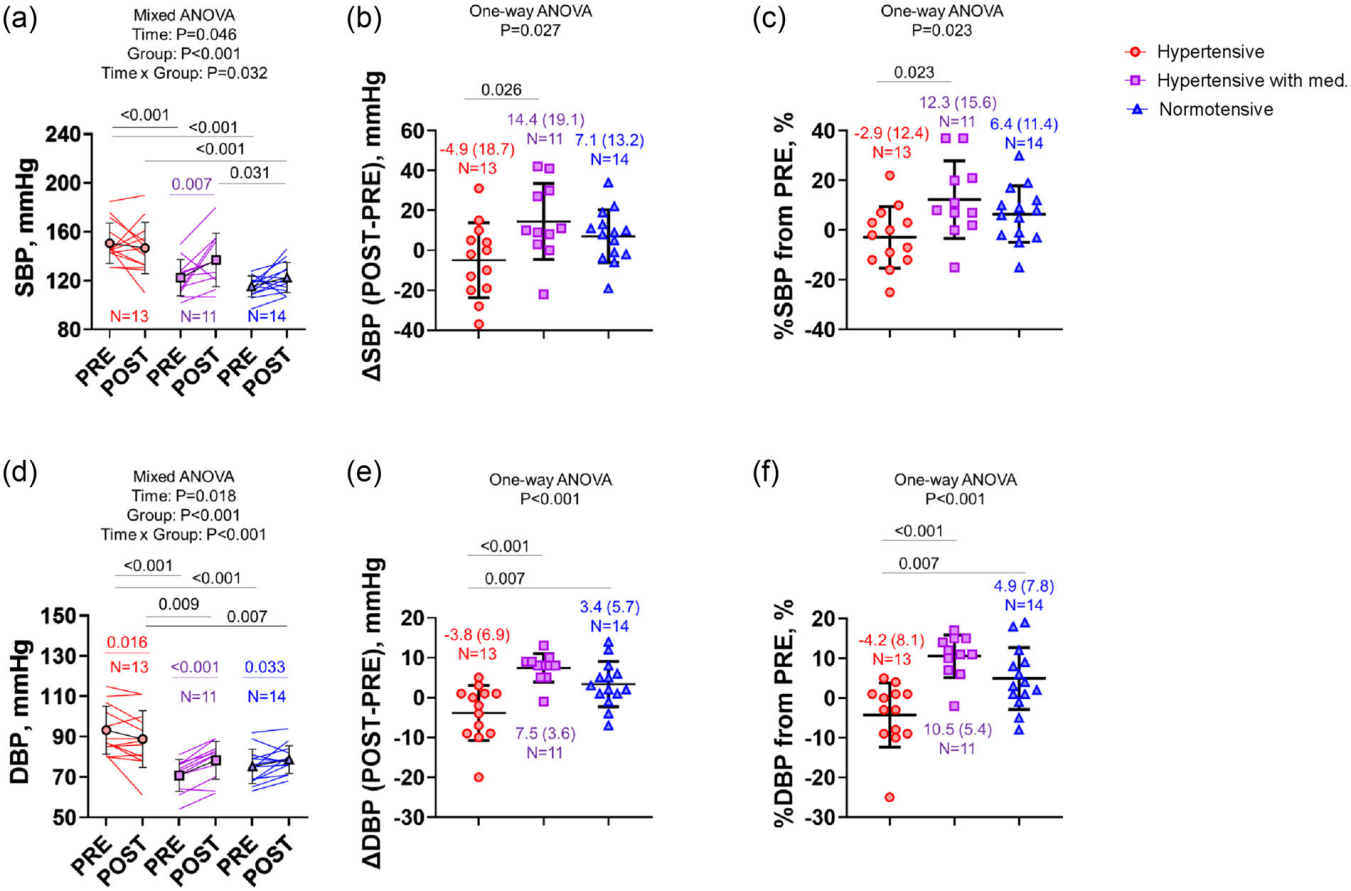
Change in systolic and diastolic blood pressure (∆SBP and ∆DBP) from pre‐ to post‐exercise (a, c) and percentage change in SBP and DBP from pre‐exercise (b, d) in untreated hypertensive (Hypertensive), treated hypertensive (hypertensive with med.) and normotensive individuals. The circles, squares and triangles denote the hypertensive, hypertensive with med. and normotensive individuals. Values are means (standard deviation). *N*, number of individuals. One untreated hypertensive participant was excluded due to missing post‐exercise BP measurements.

SBP increased from pre‐ to post‐exercise in the treated hypertensive group but not in the untreated hypertensive or normotensive groups (Figure 2a; Interaction (time × group), *P* = 0.032). Both ∆SBP from pre‐ to post‐exercise and percentage change SBP from pre‐exercise showed a greater response in the treated hypertensive group than in the untreated hypertensive group (Figure 2b,c; *P *< 0.05). DBP increased from pre‐ to post‐exercise in both the treated hypertensive and normotensive groups, while it decreased in the untreated hypertensive group (Figure 2d; Interaction (time × group), *P *< 0.001). Both ∆DBP from pre‐ to post‐exercise and percentage change in DBP from pre‐exercise showed a significantly different response between the treated hypertensive or normotensive groups and the untreated hypertensive group (Figure 2e,f; *P *< 0.001 and *P* = 0.007, respectively).

### 3.3 Motor unit firing patterns

**FIGURE 3:**
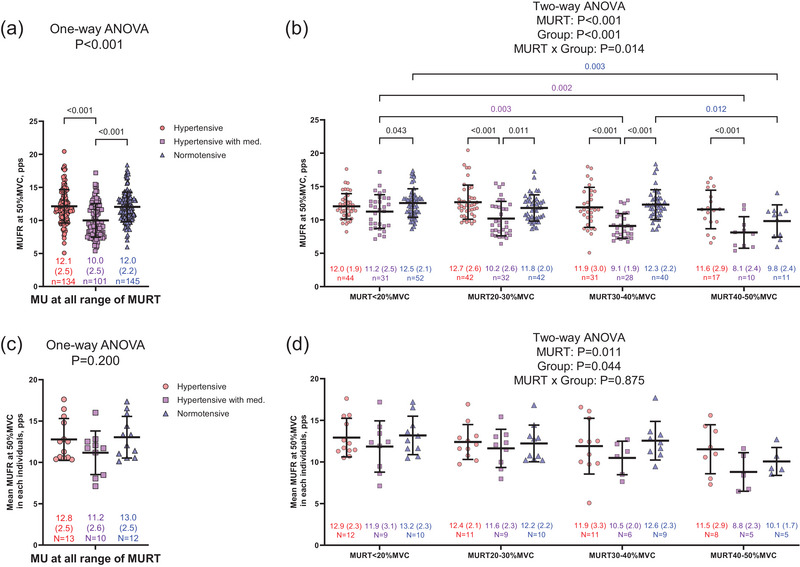
Motor unit firing rate (MUFR) and mean MUFR at 50% of maximal voluntary contraction (MVC) in untreated hypertensive (hypertensive), treated hypertensive (hypertensive with med.), and normotensive individuals. (a) Each MUFR in all MU recruitment thresholds (RT). (b) Each MUFR in each range of MURT (<20% MVC, 20–30% MVC, 30–40% MVC and 40–50% MVC). (c) The mean MUFR in all MURT from each participant. (d) The mean MUFR in each range of MURT from each individual. The circles, squares and triangles denote the hypertensive, hypertensive with med. and normotensive individuals. Values are means (standard deviation). *n*, number of MU, *N*, number of individuals. Note that some participants may have missing data due to the absence of MUs in specific or all MURT ranges.

The total number of detected MUs for the calculation of FRs was 134 in 13 untreated hypertensive, 101 in 10 treated hypertensive, and 145 in 12 normotensive individuals. MUFR at 50% MVC was significantly lower in treated hypertensive than untreated hypertensive and normotensive individuals (Figure 3a; *P *< 0.001). The FRs of larger MUs (in the range of MU recruitment thresholds between 40% and 50% MVC) were significantly lower than the FRs of smaller MUs (in the range of MU recruitment thresholds below 20% MVC) in the treated hypertensive and normotensive groups (Figure 3b; *P* = 0.002 and *P* = 0.003, respectively), but this phenomenon disappeared in the untreated hypertensive group (Figure 3b; *P* = 0.900). Although mean MUFR at 50% MVC across all ranges of MU recruitment thresholds was not significantly different between groups (Figure 3b; *P* = 0.200), the effect size between mean FR of larger MUs (in the range of MU recruitment thresholds between 40% and 50% MVC) and mean FR of smaller MUs (in the range of MU recruitment thresholds below 20% MVC) was large in the treated hypertensive and normotensive groups (Figure 3d; Cohen's *d* = 1.08 and 1.45, respectively), but medium in the untreated hypertensive group (Cohen's *d* = 0.55).

**FIGURE 4:**
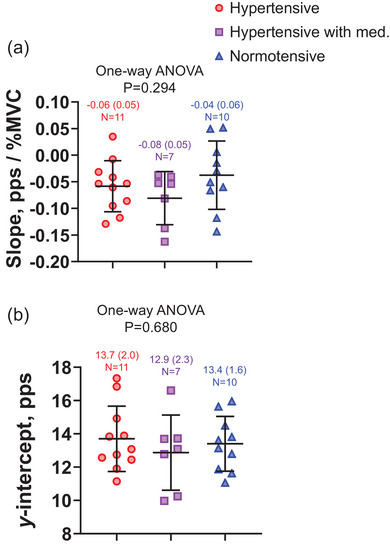
Comparison of the slope (a) and *y*‐intercept (b) of the linear regression between firing rates of individual motor units and recruitment thresholds between untreated hypertensive (hypertensive), treated hypertensive (hypertensive with med.) and normotensive individuals during ramp contraction at 50% of the maximal voluntary contraction. The circles, squares and triangles denote the hypertensive, hypertensive with med. and normotensive individuals. Values are means (standard deviation). *N*, number of individuals. Participants with fewer than five detected MU were excluded from the analysis based on criteria for the slope and *y*‐intercept of the linear regression.

The slope from MUFRs versus recruitment threshold relationships, an index of larger age‐related alterations in (higher value) or smaller age‐related alterations in (lower value) MU firing pattern, was not significantly different between groups (Figure 4a; *P* = 0.294). The *y*‐intercept, interpreted as the amount of neural input from the central nervous system, was not significantly different between groups (Figure 4b; *P* = 0.680). As shown in Table 2, the slope was not associated with the *y*‐intercept and mean FR of smaller MUs (in the range of MU recruitment thresholds below 40% MVC) but was strongly correlated with the mean FR of larger MUs (in the range of MU recruitment thresholds between 40% and 50% MVC) only in the untreated hypertensive group (*r* = 0.712, *P* = 0.048). Thus, older untreated hypertensive individuals with larger age‐related alterations in MU firing patterns relied on higher FRs of larger MUs, whereas those with relatively smaller age‐related alterations in MU firing patterns did not rely on them during exercise at 50% MVC.

**TABLE 2:** Pearson's correlation between the slope and *y*‐intercept or mean motor unit firing rate at each range of recruitment threshold.

		*y*‐intercept	Mean MUFR <20% MURT	Mean MUFR 20–30% MURT	Mean MUFR 30–40% MURT	Mean MUFR >40% MURT
**Untreated hypertensive**						
Slope	*r*	−0.037	0.235	0.396	0.281	**0.712**
	*P*	0.920	0.513	0.228	0.432	**0.048**
	*N*	10	10	11	10	**8**
**Treated hypertensive**						
Slope	*r*	−0.590	−0.387	0.010	−0.291	0.344
	*P*	0.164	0.391	0.983	0.576	0.571
	*N*	7	7	7	6	5
**Normotensive**						
Slope	*r*	−0.037	0.407	0.631	0.640	0.806
	*P*	0.920	0.317	0.0504	0.087	0.100
	*N*	10	8	10	8	5

Abbreviations: *N*, number of participants; MUFR, motor unit firing rate; MURT, motor unit recruitment threshold.

Bold values indicate significant correlations between the slope and any parameters, with corresponding *r*‐values, *P*‐values, and number of participants provided.

### 3.4 Association between change in BP from pre‐ to post‐exercise and neural components

Table 3 depicts Pearson's correlation between ΔBP from pre‐ to post‐exercise and *y*‐intercept or mean FR at each range of MU recruitment thresholds. ΔSBP was not associated with *y*‐intercept, mean FR of smaller MU (in the range of MU recruitment thresholds below 40% MVC) but was strongly correlated with mean FR of larger MU (in the range of MU recruitment thresholds between 40% and 50% MVC) only in the untreated hypertensive group (*r* = 0.768, *P* = 0.044). There was no significant correlation between ΔDBP from pre‐ to post‐exercise and any of the variables in either hypertensive or normotensive individuals.

**TABLE 3:** Pearson's correlation between the change in systolic or diastolic blood pressure from pre‐ to post‐exercise and *y*‐intercept or mean motor unit firing rate at each range of recruitment threshold.

		*y*‐intercept	Mean MUFR <20% MURT	Mean MUFR 20–30% MURT	Mean MUFR 30–40% MURT	Mean MUFR >40% MURT
**Untreated hypertensive**						
∆SBP	*r*	0.041	0.398	0.483	0.519	**0.768**
	*P*	0.910	0.225	0.157	0.124	**0.044**
	*N*	10	11	10	10	**7**
∆DBP	*r*	0.259	0.540	0.394	0.416	0.541
	*P*	0.469	0.086	0.260	0.232	0.209
	*N*	10	11	10	10	7
**Treated hypertensive**						
∆SBP	*r*	−0.504	0.023	−0.094	−0.512	0.110
	*P*	0.249	0.954	0.810	0.300	0.860
	*N*	7	9	9	6	5
∆DBP	*r*	−0.156	0.443	0.154	−0.206	−0.027
	*P*	0.739	0.233	0.693	0.695	0.965
	*N*	7	9	9	6	5
**Normotensive**						
∆SBP	*r*	−0.312	−0.134	−0.353	−0.312	−0.293
	*P*	0.380	0.712	0.317	0.414	0.632
	*N*	10	10	10	9	5
∆DBP	*r*	−0.178	0.414	0.019	0.018	−0.074
	*P*	0.624	0.234	0.958	0.963	0.906
	*N*	10	10	10	9	5

Abbreviations: ∆DBP, change in diastolic blood pressure from pre‐ to post‐exercise; ∆SBP, change in systolic blood pressure from pre‐ to post‐exercise; MUFR, motor unit firing rate; MURT, motor unit recruitment threshold; *N*, number of participants.

Bold values indicate significant correlations between ∆SBP or ∆DBP from pre‐ to post‐exercise and any parameters, with corresponding *r*‐values, *P*‐values, and number of participants provided.

**FIGURE 5:**
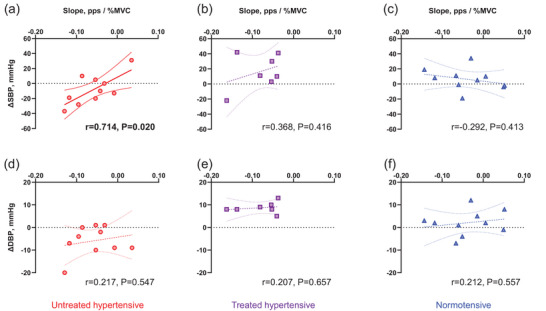
Association between the motor unit firing pattern (slope) and changes in systolic blood pressure (∆SBP) (untreated hypertensive, (a); treated hypertensive, (b); normotensive, (c)) or ∆DBP from pre‐ to post‐exercise (untreated hypertensive, (d); treated hypertensive, (e); normotensive, (f)). The circles denote the untreated hypertensive individuals (*N* = 10), the squares denote the treated hypertensive individuals (*N* = 7), and the triangles denote the normotensive individuals (*N* = 10). Pearson's correlation was used to assess the association. Note that some participants may have missing data due to the absence of ∆SBP, ∆DBP or slope. Bold values indicate significant correlations between ∆SBP or ∆DBP from pre‐ to post‐exercise and the slope, with corresponding *r*‐values and *P*‐values provided.

ΔSBP from pre‐ to post‐exercise was positively correlated with the slope in untreated hypertensive individuals only (Figure 5a; *r* = 0.714; *P* = 0.020). There was no significant correlation between ΔDBP from pre‐ to post‐exercise and any of the variables in either hypertensive or normotensive individuals.

### 3.5 Association between change in BP from pre‐ to post‐exercise and MVC or muscle components

Table 4 depicts Spearman's correlation between ΔBP from pre‐ to post‐exercise and MVC, and Pearson's correlation between ΔBP from pre‐ to post‐exercise and MT or EI. There was no significant correlation between ΔBP from pre‐ to post‐exercise and any of the variables in either hypertensive or normotensive individuals except a significant negative correlation between ΔDBP from pre‐ to post‐exercise and EI in treated hypertensive individuals.

**TABLE 4:** Pearson or Spearman's change in systolic or diastolic blood pressure and maximal voluntary contraction, muscle thickness and muscle echo intensity.

		MVC (a)	Muscle thickness	Muscle echo intensity
**Untreated hypertensive**				
∆SBP	*r*	0.418	0.032	0.341
	*P*	0.156	0.917	0.253
	*N*	13	13	13
∆DBP	*r*	0.440	−0.222	0.079
	*P*	0.133	0.466	0.798
	*N*	13	13	13
**Treated hypertensive**				
∆SBP	*r*	0.079	0.004	−0.446
	*P*	0.829	0.992	0.170
	*N*	10	11	11
∆DBP	*r*	0.283	0.212	−0.786
	*P*	0.428	0.531	0.004
	*N*	10	11	11
**Normotensive**				
∆SBP	*r*	0.022	0.274	0.039
	*P*	0.940	0.343	0.895
	*N*	14	14	14
∆DBP	*r*	−0.158	0.053	−0.088
	*P*	0.590	0.857	0.766
	*N*	14	14	14

*Note*: (a) indicates the *P*‐value and *r*‐value from Spearman's correlation.

Abbreviations: ∆DBP, change in diastolic blood pressure from pre‐ to post‐exercise; ∆SBP, change in systolic blood pressure from pre‐ to post‐exercise; MVC, maximal voluntary contraction; *N*, number of participants.

## 4 DISCUSSION

The major findings of this study were the following: (1) MUFR was lower in hypertensive individuals using ARBs than in untreated hypertensive and normotensive individuals; (2) the FRs of larger MUs were lower than those of smaller MUs in both treated hypertensive and normotensive individuals; however, the FRs between larger and smaller MUs did not differ in untreated hypertensive individuals; (3) the FRs of larger MUs, but not those of smaller MUs, were associated with the slope, an index of larger age‐related alterations in (higher value) or smaller age‐related alterations in (lower value) MU firing patterns, in untreated hypertensive, but not in treated hypertensive and normotensive individuals; and (4) post‐exercise SBP response was positively correlated with FR of larger MUs and the slope in untreated hypertensive individuals, while these relationships were not observed in treated hypertensive and normotensive individuals. These results support our hypothesis and suggest that the MU firing pattern in treated hypertensive is altered compared to that in untreated hypertensive and normotensive individuals, and the FR of larger MU and MU firing patterns are associated with the post‐exercise SBP response in older untreated hypertensive individuals. Interestingly, this relationship was not revealed in treated hypertensive as well as normotensive individuals.

### 4.1 Hypertension and motor unit firing pattern

MUFR was significantly lower in treated hypertensive individuals compared to untreated hypertensive and normotensive individuals (Figure 3a). This may be explained by the ARB‐induced neurotrophic effect on spinal motor neurons (Iwasaki et al., 2002). Indeed, the FRs of larger MUs were lower than those of smaller MUs in treated hypertensive as well as normotensive individuals, but the FRs between larger and smaller MUs did not differ in untreated hypertensive individuals (Figure 3b). Since MUs exhibit a hierarchical firing pattern based on recruitment thresholds (Watanabe et al., 2016), lower FRs of larger MUs compared to those of smaller MUs means smaller age‐related alterations in MU firing patterns. It is important to note that the group differences in MUFR disappeared when the data were averaged for each participant (Figures 3c,d; one‐way ANOVA, *P* = 0.200, and *post hoc* test between group, *P *> 0.05). This may be due to decreased sample size, as multiple data points were generated by individual participants. Thus, the results from individual MUs (Figures 3a,b) may overestimate the potential impact of ARBs in preventing age‐related changes in MU firing patterns. Future studies with larger sample sizes are required to confirm whether ARBs can effectively prevent the age‐related alterations in MU firing patterns. Additionally, we recognize the potential interaction between ARBs and calcium channel blockers (CCBs), which could influence MUFR in the treated hypertensive group. Although this warrants consideration in future research, we believe CCBs are unlikely to directly affect MU firing for the following reasons: (1) while CCBs modulate excitation–contraction coupling in cardiac and vascular smooth muscle (Lucchesi, 1989; McCall, 1987), there is no evidence suggesting that they impact excitation–contraction coupling in skeletal muscle; and (2) although CCBs can reduce iron overload‐mediated oxidative stress in renal epithelial cells (Sun et al., 2020), there is no current evidence that they affect oxidative stress in skeletal muscle. Therefore, it is likely that ARBs influenced MUFRs (Figure 3a,b), while CCBs did not directly affect them.

### 4.2 Post‐exercise blood pressure response in older hypertensive individuals

In the current study, ∆SBP from pre‐ to post‐exercise was not significantly different between older untreated hypertensive and normotensive individuals, but there was a greater increase in older treated hypertensive individuals than in untreated hypertensive individuals (Figure 2a). These results support the previous research which reported antihypertensive medication could not prevent exaggerated BP during exercise in treated hypertensive individuals (Chant et al., 2018). McGowan et al. (2011) also reported that ARBs did not reduce BP response and sympathetic nerve activity to exercise. Mitchell (2017) summarized that the exaggerated BP response to exercise is due to a greater increase in sympathetic nerve activity via the activation of muscle metaboreflex and mechanoreflex in hypertensive individuals. It is important to note that 6 out of 11 treated hypertensive individuals were using CCBs in combination with ARBs. Although we did not collect specific information on the type or duration of CCB use, some studies suggest that certain CCBs can increase resting muscle sympathetic nerve activity (MSNA) even while lowering resting BP in hypertensive individuals (Grassi et al., 2003; Inomata et al., 2014). Further research is warranted to explore whether CCBs contribute to increased post‐exercise BP through heightened sympathetic activation.

On the other hand, SBP was decreased from pre‐ to post‐exercise in 7 out of 14 untreated hypertensive, 1 out of 11 treated hypertensive, and 5 out of 14 normotensive individuals (Figure 2a). This individual difference may be explained by individual differences in muscle metaboreflex‐mediated cardiac and peripheral vascular resistance during exercise (Watanabe et al., 2014). Furthermore, Kobetic et al. (2022) reported lower sympathetic transduction in hypertensive than normotensive individuals, suggesting that higher levels of MSNA are needed to achieve the same level of vasoconstrictor tone. Another study also suggested that post‐exercise hypotension is due to a centrally mediated decrease in sympathetic nerve activity with reduced signal transduction from sympathetic nerve activation into vasoconstriction in hypertensive individuals (Floras & Senn, 1991). Floras and Senn reported that MSNA was greater in young borderline hypertensive individuals than in normotensive individuals during rest. Furthermore, BP and MSNA decreased after exercise in borderline hypertensive but not in normotensive individuals (Floras & Senn, 1991). Furthermore, Cleroux et al. (1992) reported that post‐exercise BP is reduced in untreated hypertensive but not normotensive individuals. This reduction was attributed to post‐exercise modulation of baroreflex control of forearm vascular resistance, leading to decreased vascular resistance, particularly in skeletal muscles (Cleroux et al., 1992). This mechanism may explain the smaller post‐exercise BP response observed in some hypertensive participants. In addition to this, our result provided new insight regarding another mechanism.

### 4.3 Potential mechanisms of association between post‐exercise BP response and MU firing pattern in hypertensive individuals

The larger age‐related alterations in the MU firing pattern (less negative slope) might contribute to the individual difference of ∆SBP from pre‐ to post‐exercise in older untreated hypertensive individuals (Figure 5a). The decline in the quality of MU firing pattern in older individuals involves a decrease in the inhomogeneity of muscle fibre types with ageing (Watanabe et al., 2016). In ageing muscle, the reduction in the inhomogeneity of muscle fibre types results from increased innervation ratio, reinnervation and/or the clustering of similar types of muscle fibres (Deschenes, 2004; Lexell et al., 1986; Sjostrom et al., 1986). Interestingly, Fuglevand and Segal's study showed a 14‐fold reduction in the efficiency of microvascular unit perfusion when they simulated age‐related alterations in MU recruitment (Fuglevand & Segal, 1997). Since microvascular unit perfusion during exercise becomes greater at higher force levels, it may be necessary to increase the activity of larger MUs to ensure adequate capillary perfusion during exercise in older individuals. However, activating the larger MUs also increases intramuscular pressure (Sadamoto et al., 1983), leading to increased peripheral vascular resistance (Osada et al., 2015). From our results of the relationship between post‐exercise SBP response and FRs of larger MUs (Table 3) and MU firing pattern (Figure 5a), older hypertensive individuals who can rely on lower MUs during exercise may be able to prevent an exaggerated BP response during exercise and post‐exercise, whereas those who need to rely on larger MUs during the same levels of exercise may experience an exaggerated BP response. Further studies are needed to clarify the physiological significance of this unique MU firing strategy in the older hypertensive individuals.

Individual differences in recruited muscle fibre types during exercise (Fallentin et al., 1985) may also be important. Frisk‐Holmberg et al. (1983) showed that the increase in BP in response to a 1‐min isometric handgrip at 33% MVC was positively associated with the percentage of fast‐twitch (FT) fibres in the VL muscle, especially in hypertensive individuals. These reports indicate that using FT muscle fibres contributes to increased BP in response to exercise in hypertensive individuals. Our result suggests that older untreated hypertensive individuals with a lower quality of MU firing pattern (less negative slope) relied on more firing of later recruited MUs than those with a higher quality of firing pattern, even when exerting a similar force during exercise (Table 2). On the other hand, in older treated hypertensive and normotensive individuals, the MU firing pattern was not associated with FRs of larger MUs (Table 2). This suggests that age‐related changes in MU firing patterns in these groups may not contribute to increased peripheral vascular resistance. Indeed, the association between the slope and ∆SBP from pre‐ to post‐exercise disappeared in treated hypertensive individuals. Thus, mechanisms of increase in SBP after exercise in treated hypertensive and normotensive individuals (Figure 2) may not be due to larger age‐related alterations in MU firing pattern.

However, in treated hypertensive individuals, this association may have been masked by the effects of CCBs. As mentioned earlier, CCBs can increase resting MSNA, which may affect post‐exercise BP even though they do not directly impact MUFRs. Therefore, in treated hypertensive individuals, CCB‐induced sympathetic activation may influence post‐exercise SBP increase, rather than age‐related alterations in MU firing patterns.

### 4.4 Study limitations

The current study had several limitations. First, BP was measured at the wrist. Although we carefully observed that participants maintained their wrist at heart level during BP measurement, we cannot exclude the possibility of wrist movement, which may have led to over‐ or underestimation of the data. However, in our preliminary study comparing wrist BP device and an upper arm device (HBP‐8000, OMRON, Kyoto, Japan) using 20 measurements corrected from 11 individuals, resting SBP and DBP were highly correlated between devices with similar values (wrist SBP and upper arm SBP: 117 (15) and 119 (16) mmHg, *r* ^2^ = 0.974, *P *< 0.001; wrist DBP and upper arm DBP: 74 (7) and 77 (9) mmHg, *r* ^2^ = 0.692, *P *< 0.001). The ∆SBP from pre‐ to post‐exercise also indicated a good correlation (∆wrist SBP and ∆upper arm SBP: 2 (8) and 1 (6) mmHg, *r* ^2^ = 0.705, *P *< 0.001), whereas ∆DBP from pre‐ to post‐exercise indicated no significant correlation (∆ wrist DBP and ∆ upper arm DBP: 3 (6) and 1 (6) mmHg, *r* ^2^ = 0.164, *P* = 0.076). Second, BP was measured only once before and after exercise. Single measurements may not fully capture fluctuations in BP and could be influenced by the unusual environment, potentially leading to overestimation. More importantly, the rate of BP recovery following exercise likely differs between individuals, meaning that although post‐exercise measurements were taken at the same time for all participants, the BP readings may have been captured at different points in each individual's recovery process. This variability could account for the heterogeneity observed in post‐exercise BP responses. Furthermore, BP was not measured during exercise. To clarify the relationship between MU firing patterns during exercise and the pressure response, BP should be measured during exercise in future studies. Third, although we speculated on how sympathetic nerve activity may have affected our results, we did not assess heart rate, heart rate variability or MSNA to evaluate the impact of the sympathetic neural response. Thus, future studies are warranted to assess the mechanisms underlying the post‐exercise BP response in older hypertensive individuals with or without antihypertensive treatment and normotensive individuals.

### 4.5 Conclusion

Our findings suggest that the MU firing pattern was different between hypertensive individuals with ARB use and untreated hypertensive or normotensive individuals. Furthermore, the age‐related decline in the MU firing pattern is associated with greater FRs of larger MUs and the SBP response from pre‐ to post‐exercise in older untreated hypertensive individuals. However, this phenomenon was not observed in hypertensive individuals using ARBs or in normotensive individuals. We suggest that, although the MU firing patterns do not differ among the three groups, the contributing factor to the individual differences in MU firing patterns varies between untreated hypertensive individuals and others. Older untreated hypertensive individuals with greater age‐related alterations in MU firing patterns rely more on the firing of larger MUs, potentially leading to greater twitch force of predominantly FT muscles. This results in a greater increase in peripheral vascular resistance and post‐exercise BP response. On the other hand, older untreated hypertensive adults with fewer age‐related alterations in MU firing patterns do not rely on the firing of larger MUs and may be able to prevent an increased post‐exercise BP response.

## AUTHOR CONTRIBUTIONS

Ryosuke Takeda obtained funding support and contributed to the analysis and interpretation of data, drafting the article, and revising it critically for important intellectual content. Ryosuke Takeda, Tetsuya Hirono, Akito Yoshiko, Shun Kunugi, Masamichi Okudaira, Saeko Ueda, and Taichi Nishikawa contributed to the conception and design of the experiments, collection, analysis, and interpretation of data, and revising the article for important intellectual content. Kohei Watanabe obtained funding support, contributed to the collection, analysis, and interpretation of data, and also contributed to drafting the article and revising it critically for important intellectual content. All authors have read and approved the final version of this manuscript and agree to be accountable for all aspects of the work in ensuring that questions related to the accuracy or integrity of any part of the work are appropriately investigated and resolved. All persons designated as authors qualify for authorship, and all those who qualify for authorship are listed.

## CONFLICT OF INTEREST

None declared.

## Data Availability

The data that support the findings of this study are available from the corresponding author upon reasonable request.
